# A Tale of Two Pandemics: Economic Inequality and Support for Containment Measures in Peru

**DOI:** 10.1177/1866802X211035393

**Published:** 2021-12

**Authors:** Miguel Carreras, Sofia Vera, Giancarlo Visconti

**Affiliations:** 1 8790 Department of Political Science, University of California Riverside, CA, USA; 2 123982 Department of Political Science, University of Kansas, Lawrence, KS, USA; 3 311308 Department of Political Science, Purdue University, West Lafayette, IN, USA

**Keywords:** Peru, inequality, attitudes and behaviour, COVID-19, Latin America

## Abstract

Research suggests that the coronavirus pandemic disproportionately affected poor communities. However, relatively little is known about how this differential impact affected support for, and compliance with, COVID-19 lockdown policies. This article examines the relationship between socioeconomic inequalities and public opinion towards COVID-19 containment measures in Peru. Despite the strict quarantine measures adopted by the government of Peru, the country struggled to contain the spread of the disease. We designed and implemented a nationally representative survey in Peru and found that economically vulnerable sectors are more likely to oppose the quarantine and are more likely to defy the stay-at-home recommendations to leave home and go to work. Our contribution highlights that poor citizens’ housing and economic conditions can explain why the poor are more likely to react negatively to COVID-19 lockdown policies.

## Abstract (es)

nvestigaciones previas sugieren que la pandemia del coronavirus afectó de manera desproporcionada a las comunidades más pobres y vulnerables socioeconómicamente. Sin embargo, se sabe relativamente poco acerca de cómo este impacto diferencial afectó al apoyo y cumplimiento de las políticas de mitigación del COVID-19. Este artículo examina la relación entre las desigualdades socioeconómicas y el apoyo de la opinión pública a las medidas de contención del COVID-19 en Perú. A pesar de las estrictas medidas de cuarentena adoptadas por el gobierno, Perú experimentó grandes dificultades para contener la propagación de la enfermedad. Diseñamos e implementamos una encuesta representativa a nivel nacional en Perú y encontramos que los sectores económicamente vulnerables tienen más probabilidades de oponerse a la cuarentena y es más probable que desafíen las recomendaciones de quedarse en casa para poder salir a trabajar. Nuestra contribución destaca que las condiciones económicas y de vivienda de los ciudadanos explican por qué es más probable que los grupos menos privilegiados reaccionen negativamente a las políticas de mitigación del COVID-19.

## Introduction

In the early days of the pandemic, the COVID-19 disease was sometimes described as an “equal opportunity offender” (Bainbridge, 2020) because people of all backgrounds could be infected and wealthy countries could suffer large outbreaks. However, it soon became clear that the most economically vulnerable sectors of society suffered disproportionately from the pandemic in every country.

Not only are the poor more likely to become infected by COVID-19, but they are also at much greater risk of suffering devastating economic consequences from the pandemic and the containment measures adopted to combat it. The more economically vulnerable groups are more exposed to these combined risks as a result of several factors, including precarious frontline jobs, crowded housing, poor access to public services, and lack of savings.

While the poor suffer disproportionately from COVID-19 everywhere, we surmise this differential impact is exacerbated in developing areas, and especially in contexts of high economic inequality and labour informality. Poor informal workers in developing countries have no access to unemployment or healthcare benefits, and they cannot afford to stay home to prevent infection because they often lack savings. Moreover, the types of jobs they have cannot be performed remotely. Abiding by public health recommendations to stay home during the early phase of the pandemic could result in hunger and extreme poverty for these vulnerable groups.

In this article, we study the differential impact of COVID-19 in the developing world by focusing on the case of Peru, a Latin American country that suffered one of the worst outbreaks in the world. Peru provides an excellent setting for this research for three reasons. First, Peru struggled to contain the spread of COVID-19 despite the strict quarantine measures adopted by the government early on in the pandemic. Second, non-compliance was one of the main challenges the government faced when attempting to reduce contagion risk. Reports about crowding in popular markets and bus stations in impoverished areas of the country raised the alarm about the ineffectiveness of the lockdown in low-income communities. Finally, the socioeconomic inequalities in Peru, as in other Latin American countries, are such that less privileged groups may face greater obstacles to abiding by the quarantine measures. These conditions make Peru a suitable country for studying the relationship between socioeconomic inequality and COVID-19 public opinion.

Our goal in this study is twofold. First, this article provides descriptive evidence of the several ways in which COVID-19 had a disproportionate negative impact on more economically vulnerable sectors of the population. Second, the article explains how this differential impact affected attitudes about (and compliance with) containment measures and social distancing guidelines. Based on theories that highlight the role of self-interest in shaping policy attitudes and behaviours (Doherty et al., 2006; Downs, 1957), we expect individuals in an economically vulnerable situation to react negatively to COVID-19 lockdown policies. The adverse reaction of low-income groups, we argue, includes lower support for lockdown measures and weaker compliance with stay-at-home recommendations.

To understand how socioeconomic inequality affects attitudes and behaviours towards COVID-19 lockdown policies, we implemented a nationally representative survey by telephone at the end of May 2020. As the key independent variable, we use an indicator that captures socioeconomic vulnerabilities from a multi-dimensional perspective by including questions about education and access to crucial goods and services such as internet and private health insurance. Meanwhile, our dependent variables capture people’s preferences about health measures and their willingness to do certain activities in the near future. We use a linear probability model with fixed effects at the department level to explore how socioeconomic vulnerabilities predict these outcomes of interest.

Our results show that Peruvians who are economically vulnerable are less likely to support quarantine measures and more likely to support the reopening of the economy than those who are economically privileged. We also find that the willingness to comply with stay-at-home guidelines depends on the level of economic necessity: less privileged citizens are more likely to ignore stay-at-home recommendations and leave home to go to work. That said, they are not more likely to engage in leisure activities outside of the home than the more privileged citizens.

These findings make two contributions. First, the results are consistent with the theoretical arguments that emphasise the role that material conditions play in shaping policy attitudes and behaviours (Becker, 1993; Cialdini, 1991; Kim, 2014). This article, thus, provides novel evidence from a different set of policy preferences during a public health emergency that is consistent with such approaches. Second, this article provides some individual-level evidence of public opinion towards quarantine measures that complements existing studies showing that poor communities were hardest hit by the health and economic consequences of the pandemic. In recent studies, scholars have argued that one of the reasons for the ineffectiveness of lockdowns in low-income communities was the economic and social inequalities that affected how poor and rich experience the pandemic (Bennett, 2021; Hummel et al., 2021). For instance, Hummel et al. (2021) show that poor departments in Bolivia had worse health outcomes and relaxed stay-at-home orders earlier. This is due, in part, to the low state capacity and the low-quality public services that exist in the poorer departments (Hummel et al., 2020), but also to the lower ability of people living in poor areas to abide by stringent public health recommendations. Similarly, Bennett (2021) demonstrates that in poor areas of Chile’s capital city quarantine effectiveness was lower than in rich areas. But there has been little individual-level evidence to support the assumption that more economically vulnerable individuals have weaker support for lockdown policies. We fill this gap by showing that both support for and compliance with lockdown policies, at the individual level, are affected by socioeconomic conditions.

## The Disproportionate Effect of the Pandemic on Low-Income Groups

The impact of the COVID-19 pandemic on employment and wages is highly unequal and exacerbates existing inequalities. Studies conducted in economically advanced OECD countries reveal that low-skilled, less educated, and low-income workers were more likely to lose their jobs or to suffer a drop in earnings as a result of the public health crisis. Work arrangements also mattered. More specifically, employees with permanent contracts were considerably less likely to lose their jobs than workers with alternative work arrangements. Moreover, workers in occupations that cannot be performed remotely were more likely to see their working hours reduced (Adams-Prassl et al., 2020; Montenovo et al., 2020).

The disparate impact of the pandemic on different socioeconomic groups was mitigated in OECD countries by the adoption of generous emergency programmes to help the most economically vulnerable groups. These policies included increasing unemployment benefits, distributing stimulus checks, or subsidising companies so that they could maintain their workforces (Birnbaum, 2020). Such large-scale policy interventions are not available in developing countries, which can lead to greater and more rapid negative effects on the economic well-being of large segments of the population (Evans and Over, 2020).^1^

There are three factors that make the negative effects of the pandemic on the economic well-being of the more vulnerable sectors of the population much more acute in developing countries.

First, there are large inequalities in access to digital networks and in the skills required to use computerised networks optimally. While access to internet is widespread in economically advanced countries, digital inequalities create a major vulnerability to the sanitary and economic consequences of COVID-19 in low- and middle-income countries (Beaunoyer et al., 2020; Robinson et al., 2015; Sambuli, 2016). People who lack reliable access to computers and reliable internet access in their houses are not able to work remotely. They are also much less able to socially isolate since they need to leave their houses to engage in essential activities such as grocery shopping and banking. Moreover, the lack of internet access makes it harder for people to maintain social contacts during lockdown periods, which can have detrimental effects on mental health (Beaunoyer et al., 2020; Guitton, 2020). In sum, the COVID-19 pandemic exacerbates already existing digital inequalities and exposes the most economically vulnerable sectors to considerably greater economic and health risks.

Second, developing countries (and Latin American countries in particular) tend to have large informal sectors (Gasparini and Tornarolli, 2009; Portes and Hoffman, 2003). The informal segment of the labour market is made up of non-professionals, unskilled labourers, marginal workers, the self-employed, domestic and family workers, and workers in small firms – all of whom engage in labour activities that are not regulated by the state and lack access to the social security system or pension system (Hussmans, 2004; Saavedra and Chong, 1999). Informal workers are less protected against the vicissitudes of professional life even during normal times since they lack “access to protection against health and unemployment shocks, to savings for old age, to employment protection and to labour related benefits” (Tornarolli et al., 2014: 3). It is therefore not surprising that they also suffer much more acutely from the COVID-19 pandemic. To begin with, informal workers (e.g. street vendors, shopkeepers, and domestic workers) have jobs that simply cannot be done remotely (Hummel et al., 2020: 120). Not going to work for a few weeks as a result of a government-imposed lockdown might mean that informal workers do not receive any form of compensation during that period. Moreover, informal workers do not have unemployment benefits when they lose their jobs. While some governments in the developing world implemented emergency programmes to provide cash assistance to the population during lockdowns, informal workers are harder to reach because they are not on state payrolls. This can generate important delays in the disbursement of funds, which can lead to acute economic distress among informal workers.

Third, the housing and living conditions of vulnerable economic groups in developing countries are often dire, which exacerbates the health risks associated with COVID-19. In particular, poor families tend to suffer from overcrowding and a lack of amenities (Rondinelli, 1990; Tipple and Willis, 2004). Crowded housing implies that several generations live together in small homes in very dense communities. Public health recommendations to maintain social distance and isolate the elderly ring hollow in these housing conditions (Hummel et al., 2020: 121). Moreover, in low-income communities, people often lack basic amenities such as a refrigerator or private sanitation. Again, those housing deficiencies make it very hard for the poor to stay home and maintain the recommended social distance since they have to go out several times a day to buy food and use the toilet.

In sum, the pandemic’s disproportionate effect on the most economically vulnerable individuals is exacerbated in developing nations. This disparity is partly due to three realities: digital inequalities, the large informal sector, and poor living conditions. These structural characteristics of developing nations were undoubtedly present in Peru when the pandemic hit. In the following section, we provide descriptive evidence of how the living and working conditions of poor Peruvians differ from those of more economically privileged Peruvians. The nature of these socioeconomic inequalities in Peru, we will argue, results in vastly different attitudes and behaviours towards the COVID-19 policies adopted by the Peruvian government.

## Inequalities in Peru

The pandemic struck the poor the hardest in Peru. When the WHO declared COVID-19 a global pandemic, the government of Peru adopted some of the swiftest and strictest containment measures in the region. The national quarantine banned people from leaving their homes, except for essential trips. Yet remaining at home required living conditions that less privileged Peruvians do not enjoy, such as adequate housing, access to water, and sanitation. Quarantining also required workers to perform their jobs from home. However, many Peruvians lack access to internet or a computer at home and have informal jobs in occupations that rarely lend themselves to remote work. In this section, we document the inequalities in housing and working conditions that made quarantining a difficult feat in Peru, especially for the poor.

The Peruvian government introduced harsh and early measures to stop the spread of the virus. A state of national emergency was announced in mid-March, just a few days after the country’s first coronavirus case was confirmed. It established a nationwide quarantine that included mandatory social isolation, restriction of movement, and a ban on public gatherings. The mobility constraints were enforced by the police in the first few weeks,^2^ but by late April the government started a gradual – albeit erratic – process of relaxing the national quarantine. A crucial step towards this flexibilisation arrived on 2 May, when the president announced a plan to re-open the economy in four phases. Throughout this time, however, the disadvantaged citizens suffered the most. A detailed chronology of the changing characteristics of the quarantine mandate in Peru can be found in Appendix A in supplemental material.

Compliance with quarantine measures required living and working conditions that economically vulnerable Peruvians cannot afford. While the lockdown’s main goal was to reduce the infection rate, the policy did not take into account problems with overcrowded housing. Peruvian families frequently share the same dwelling unit with other families. Overcrowding has been associated with the rapid transmission of respiratory diseases, and it can negatively impact mental health. Even though household crowding has decreased over the last decade in Peru, the gap between the poor and the rich has remained large. An analysis of the 2019 Peruvian Household Survey (ENAHO) indicates that overcrowding is 12 percentage points higher among the population living in extreme poverty than among the non-poor.^3^

There is also a large gap in access to basic services and amenities at home. Access to sanitation facilities and drinking water in the house is still a privilege that many Peruvians do not enjoy, especially the poor. A simple analysis of the 2018/19 AmericasBarometer survey data^4^ shows that 68.4 per cent of Peruvians in the lowest income group have a toilet inside the house, compared to 94.9 per cent in the top income group. Access to water at home is also uneven. There is a 10 per cent difference in water access between the top and the bottom income groups. Finally, having a refrigerator at home to store food is also much more frequent among the rich. In the lowest income category, only 44.3 per cent of respondents have a refrigerator, whereas 90.6 of individuals in the highest income category have a refrigerator. These differences suggest that the poor suffered the most when the Peruvian government imposed restrictions on movement, as they did not have adequate housing and services.

In addition to inadequate living conditions, the working circumstances in Peru also make quarantining a difficult task that only a few can accomplish. About 68.5 per cent of Peruvians are employed in informal jobs with precarious labour arrangements, a rate that is disproportionately higher among the poorest Peruvians. We show in Appendix B in supplemental material that 89.9 per cent of employed Peruvians in the lowest income group work informal jobs, whereas employment informality among the highest income group drops to 45.9 per cent. Finally, having access to internet and a computer became essential to navigate the quarantine. Yet, only 37.5 per cent of Peruvians have access to the internet and 40.8 per cent own a computer (see Appendix B). Here, there are also stark socioeconomic gaps: in the top income group, 72.4 per cent have access to the internet and 77.2 own a computer at home, while in the bottom income group, only 11.5 per cent have internet and 13.4 per cent own a computer. This technology gap made it more challenging to accept mobility restrictions, as it effectively deprived a large population of the only financial lifeline they had.

## Economic Vulnerability, Policy Attitudes, and Social Distancing

We have shown that different socioeconomic groups in Peru face completely different realities, and these distinctions can have a direct impact on how these groups experience the pandemic. On the one hand, more privileged individuals have protected jobs, can work remotely, shop online for groceries and other essentials, and have basic amenities in their dwellings. On the other hand, more economically vulnerable people (and in particular, poor informal workers) rapidly suffer devastating consequences because they are unable to work or engage in other essential activities (e.g. banking or grocery shopping) remotely. In this section, we discuss how these different realities might have shaped people’s attitudes towards COVID-19 containment measures and their willingness to engage in a number of social and professional activities during the first wave of the COVID-19 pandemic in Peru.

We argue that Peruvians reacted to COVID-19 containment measures by considering the costs and benefits associated with those measures for them and for their families. This argument builds on a vast literature in psychology, economics, and political science that has demonstrated that self-interest is a powerful motivator of policy attitudes and human behaviours (Becker, 1993; Cialdini, 1991; Downs, 1957). Self-interest can be defined as “the motive to maximise material resources and to minimise harm to one’s wealth and health” (Kim, 2014: 100). This definition juxtaposes the two elements (material wealth and health) that were threatened by the COVID-19 pandemic. In fact, policy and media communications often framed COVID-19 containment policies as presenting a trade-off between public health and economic well-being (Carreras et al., 2020; Deslatte, 2020; Hargreaves Heap et al., 2020).

We postulate that people with different socioeconomic statuses weighed material and health considerations differently when forming their views on restrictive quarantine measures during the first wave of the COVID-19 pandemic. People who are in a position of relative economic privilege (i.e. formal white-collar workers with higher incomes) were able to maintain a steady source of income by working remotely. They also enjoyed basic amenities in their homes such as internet services, running water, and sanitation, which are critical to be able to stay at home during a prolonged lockdown period. Given the fact that their basic material needs were secure during this period, more privileged sectors of the Peruvian population may have reacted more favourably to strict quarantine measures. In all likelihood, this population group was primarily concerned with the rapid spread of a new disease with no known cure or effective treatment.

While clearly not oblivious to the health risks posed by COVID-19, the attitudes of more economically vulnerable groups towards COVID-19 containment measures may have been shaped more strongly by material concerns. As detailed in the previous section, poor informal workers in Peru cannot work remotely and they can suffer dire economic consequences if they don’t go to work every day. Economically vulnerable groups in developing countries also lack savings to overcome these economic challenges. In the words of a street vendor in Mexico City, “people with money can stay one, two, even three months at home […] but those of us who live day-to-day have no financial support” (Sheridan, 2020). The result is that lockdown measures adopted to fight the COVID-19 pandemic can lead to extreme poverty and hunger among these fragile sectors of the population. We argue that these material considerations shape the attitudes of vulnerable economic groups towards quarantine measures. In particular, we expect that these sectors of the Peruvian population are less likely to endorse lockdown measures.

This theoretical expectation builds on a large literature that has demonstrated that self-interest shapes the policy attitudes of individuals with different socioeconomic status on a range of issues, including welfare programmes (Baslevent and Kirmanoglu, 2011), trade (Fordham and Kleinberg, 2012), taxation (Hammar et al., 2008; Hennighausen and Heinemann, 2015), immigration (Nteta, 2013), and housing policies (Marble and Nall, forthcoming). For instance, people with a low socioeconomic status tend to prefer higher levels of wealth redistribution (Rueda and Stegmueller, 2019) and have less favourable views on low-skilled immigration (Scheve and Slaughter, 2001).

Other studies have challenged these self-interest explanations by showing that symbolic predispositions (acquired through socialisation) can trump self-interest in attitude formation (Kinder and Sears, 1981; Lau and Heldman, 2009). This is especially true when there are partisan cues that frame issues in a way that bring these symbolic predispositions to the forefront of individuals’ considerations (Sears and Funk, 1991; Sears et al., 1980). In the United States, lockdown measures and mask mandates adopted to fight the spread of COVID-19 were often framed as a violation of people’s freedom by the conservative media and some Republican leaders. Given the importance of symbolic predispositions emphasising freedom from government intervention among Republicans, this framing led to a partisan gap in attitudes towards (and compliance with) COVID-19 public health guidelines in the United States (Allcott et al., 2020; Gadarian et al., 2021; Utych, 2021). Calvo and Ventura (2021) report a similar finding in Brazil, where supporters of President Bolsonaro (a right-wing politician who declared the pandemic a hoax) were less likely to perceive the COVID-19 pandemic as a risk to their health.

In Peru, there is no clear cultural or symbolic predisposition that shaped views on containment measures to fight the pandemic. Unlike in the aforementioned cases, no political cleavage emerged on this issue; rather, a political consensus rapidly emerged regarding the importance of complying with public health recommendations. Moreover, the COVID-19 pandemic was a new issue that people had not encountered in the past (that is, no preconceived notions on how to best respond to a pandemic). As a result, we expect that self-interest played a very important role in shaping people’s attitudes towards quarantine measures and social distancing recommendations.

Beyond the novelty of this policy issue, we also expect self-interest to trump symbolic predispositions in most developing countries because the costs associated with lockdown measures were clear and immediately felt by individuals living in a situation of economic vulnerability. Previous research has demonstrated that self-interest plays a key role in shaping attitudes when people can easily identify how they are affected by a policy (Chong et al., 2001; Green and Gerken, 1989; Sears and Funk, 1990). This is especially true in the economic arena. In the words of Kim (2014: 109), “policies that affect voters’ pocketbooks have shown clear self-interest effects, presumably because voters can easily understand that they are financially affected by those policies and easily calculate a cost-benefit analysis of the passage of those policies.” Clearly, COVID-19 lockdown measures are a good example of such a high-stakes policy since they directly and immediately affected the livelihood of individuals with a low socioeconomic status. This discussion yields the first hypothesis of the article.

**Hypothesis 1:** Individuals in a situation of economic vulnerability are less likely to support lockdown measures to stop the spread of COVID-19.

Attitudes towards costly quarantine measures matter because policy attitudes can shape behaviour. Diminished support for quarantine under economic distress can lead to lower compliance with preventive measures (Hargreaves Heap et al., 2020). More specifically, we expect that people with a low socioeconomic status were more willing to leave their homes to work during the first months of the COVID-19 pandemic, despite the uncertainty surrounding the new illness. A study conducted in Israel (an OECD country) shows that compliance with quarantine measures was significantly lower among people who were concerned about loss of income (Bodas and Peleg, 2020). As detailed above, poor informal workers in developing countries have an even stronger incentive to go out to work during the pandemic to maintain their livelihood. However, we argue that this non-compliance emerges out of economic necessity rather than lack of concern with the severity of the public health crisis. We therefore do not expect significant differences in the willingness to engage in social activities (e.g. eating out or meeting friends) between economically privileged and economically fragile sectors of the Peruvian population during the first wave of the COVID-19 pandemic.

**Hypothesis 2:** Individuals in a situation of economic vulnerability were more willing to go out of their houses to work during the first wave of the COVID-19 pandemic.

**Hypothesis 3:** Individuals in a situation of economic vulnerability were *not* more willing to go out of their houses to engage in social activities during the first wave of the COVID-19 pandemic.

## Socioeconomic Conditions and COVID-19

To understand the differential impact of the pandemic on people’s attitudes towards COVID-19 measures in Peru, we implemented a nationally representative survey by telephone between 21 May and 28 May 2020. Our sample is composed of 1,490 respondents across the entire country. The survey included socioeconomic variables and COVID-related questions.

Peru is an ideal place to study attitudes and preferences towards COVID-19 policies because the government implemented strict measures early on, which were very difficult to enforce. In fact, to avoid discouraging compliance, the government updated the national quarantine characteristics every two weeks (as the timeline in Appendix A shows). By the time we implemented our survey in late May, the government had announced a plan for re-opening the economy. The restrictions on movement thus had begun to loosen up, and compliance with public health recommendations had become voluntary.

Our key independent variable captures the socioeconomic vulnerabilities in Peru, to then be able to check whether people who are less privileged than others have different attitudes regarding measures to stop or contain the spread of the virus. Instead of just using one single question to measure socioeconomic vulnerabilities, we use an indicator constructed by the Instituto de Estudios Peruanos that uses a weighted average of key questions such as educational level and access to goods and services such as the internet, a computer, a bathroom, and private health insurance (see Appendix D in supplemental material for more details). This variable allows us to capture the multi-dimensionality of socioeconomic vulnerability by paying attention to the different factors that can explain it, such as a lack of health insurance, internet, or a bathroom. This socioeconomic indicator uses values from 1 to 8, so we standardise it to express its changes in standard deviation units, thus facilitating the interpretation of the main analyses.

In the case of the dependent variables, we use multiple questions that allow us to capture diverse dimensions of the sanitary and economic problems that Peru was facing with the spread of COVID-19. The first set of questions captures people’s preferences: support for the quarantine, support for re-opening the economy, and support for re-opening gyms.^5^ The second set of questions measures people’s willingness to do certain activities in the near future:^6^ to go out to work, to attend a religious service, to meet up with friends, to go to the mall, and to eat out. All of the dependent variables have a binary structure facilitating the interpretation of the results and comparability across outcomes.^7^

**Figure 1:**
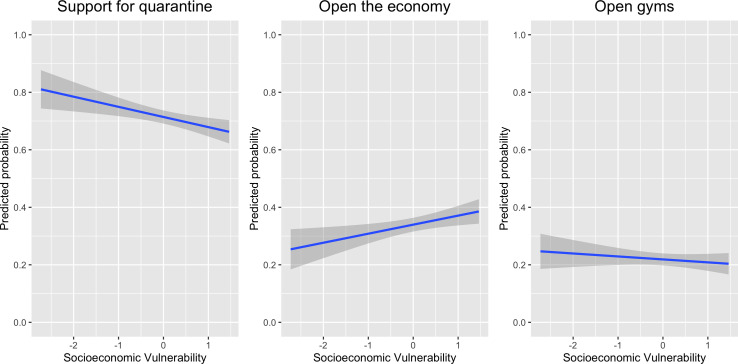
Predicted Probabilities of Policy Preferences.

To learn about differential attitudes across levels of socioeconomic vulnerabilities in Peru, we use a linear probability model with fixed effects at the department level. In addition to socioeconomic vulnerability, we include a binary indicator of health vulnerabilities for COVID-19 in the household as an important control variable.^8^ After implementing this model, we estimate the predicted probability of supporting COVID-19 containment measures across the different values of the standardised socioeconomic vulnerability variable, which ranges from −2.73 to 1.46 standard deviation units. Figure 1 summarises the results for respondents’ preferences.

We find that an increase by 1 standard deviation in socioeconomic vulnerability decreases support for quarantine by 3.22 percentage points [95 per cent CI: –5.68, –.76]. Similarly, a one-point increase in socioeconomic vulnerability actually increases support for opening the economy by 3.60 percentage points [95 per cent CI: 1.01, 6.18]. We do not find evidence of a relationship between socioeconomic vulnerability and support for opening gyms [95 per cent CI: −2.38, 2.14]. These results are consistent with hypothesis 1, positing that individuals in a socioeconomically vulnerable situation are less likely to support lockdown measures. These preferences are likely to emerge because these populations do not have the working or housing conditions to withstand the lockdown. Their basic material needs at home are not met, and they cannot maintain a regular family income by working remotely, given the limited access to internet and a computer.

**Figure 2:**
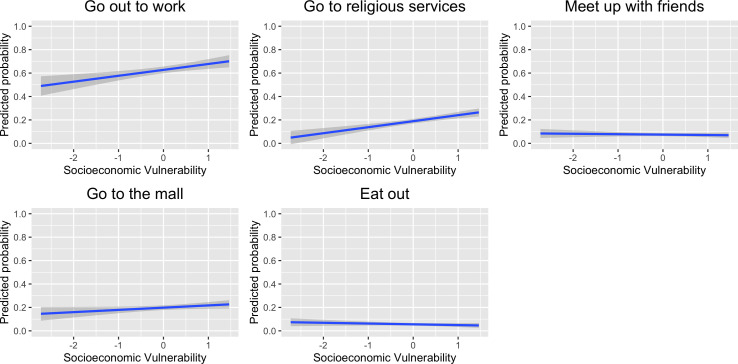
Predicted Probabilities of Willingness to Engage in Activities.

Figure 2 summarises the results for people’s willingness to do certain activities after the lockdown. We find that an increase by 1 standard deviation in socioeconomic vulnerability increases a willingness to go out to work by 3.91 percentage points [95 per cent CI: 0.80, 7.03] and a willingness to go to religious services by 4.52 percentage points [95 per cent CI: 2.41, 6.64]. We did not find evidence for a willingness to meet up with friends [95 per cent CI: −2.16, 0.71], go to the mall [95 per cent CI: −1.11, 3.24], or eat out [95 per cent CI: −2.10, 0.41] in the next few weeks. These results are consistent with our theoretical expectations that people in a situation of economic vulnerability are more likely to ignore stay-at-home recommendations because of economic necessity. In line with hypothesis 2, we observe that economically vulnerable individuals report a greater willingness to leave home to go to work than economically privileged ones. In contrast, in line with hypothesis 3, we find no evidence of non-compliance when it comes to social activities unrelated to work. The economically vulnerable are as likely as the economically privileged to skip or postpone social activities (i.e. eating out, going to the mall, or meeting up with friends). Compliance with these social activities might be a realistic option for economically vulnerable individuals because their financial lifeline does not depend on them. An interesting exception emerges concerning attending religious services. In contrast to hypothesis 3, economically vulnerable individuals are, in fact, more willing to defy public health recommendations to attend a religious service. This unexpected finding is probably due to the fact that religiosity is stronger among poor citizens (Herzer and Strulik, 2017).

## Discussion and Conclusion

The COVID-19 pandemic obliged governments in different parts of the world to adopt strict containment measures, such as curfews or lockdowns. Enforcing these measures is difficult and costly. To a large extent, governments had to rely on public support for lockdowns and people’s voluntary adherence to public health guidelines. Previous studies have demonstrated that factors such as age, gender, education, ideology, and partisanship shape compliance with COVID-19 measures (Allcott et al., 2020; Brouard et al., 2020; Calvo and Ventura, 2021; Carreras et al., forthcoming; Gadarian et al., 2021).

In this article, we show that socioeconomic status is a critical factor shaping support for containment measures and compliance with stay-at-home orders in Peru, a country with a high level of economic inequality. In particular, the results show that economically vulnerable individuals (i.e. poor and informal workers) were less likely to endorse strict lockdown measures in the early stages of the COVID-19 pandemic. Similarly, people with a low socioeconomic status expressed a greater willingness to leave their homes to go to work despite the public health emergency.

We argue that the attitudinal and behavioural differences between the haves and the have-nots are due to digital inequalities and poor housing conditions (e.g. a lack of sanitation and overcrowding) that make long periods of confinement much harder to bear for low-income individuals in developing countries. The willingness of people with a low socioeconomic status to leave their houses to go to work is connected with the fact that most of them have informal jobs that cannot be performed remotely. Moreover, not going to work can very quickly put them in dire financial straits because they lack savings and the benefits associated with jobs in the formal economy.

We do not think, however, that economically vulnerable people are less concerned or less informed about COVID-19. There is no reason why they should be. In fact, our results indicate that people with a low socioeconomic status were not more likely than wealthy individuals to engage in enjoyable social activities that were not financially rewarding (e.g. meeting up with friends or eating out) during the first months of the COVID-19 pandemic. This strongly suggests that economic considerations are the primary driver of the attitudinal and behavioural differences uncovered in this article.

While our article focused on Peru, other studies and journalistic accounts confirm that throughout Latin America the poor suffered disproportionately from the effects of lockdowns and were less likely to comply with stay-at-home orders (Ioris, 2020; Levy Yeyati and Malamud, 2020; Sandin, 2020). For instance, Hummel et al. (2021) show that labour informality and economic inequities in Bolivia led to a relaxation of stay-at-home orders and more severe COVID-19 outbreaks in the poorest departments. In a similar vein, Bennett (2021) demonstrates that quarantine compliance and effectiveness was lower in poor areas in Chile. Finally, Rodrigo Zarazaga (2020) (an Argentinian priest and political scientist) commented that the long period of lockdown in Argentina shuttered the income of independent and informal workers (almost one half of the Argentinian working population). He also claimed that “overcrowding conditions, lack of public services, precarious health systems, and food shortages makes social distancing almost an impossible mission in shantytowns and poor neighbourhoods” (Zarazaga, 2020).

The differential impact of COVID-19 containment measures (and of the pandemic itself) in Latin America raises important policy questions. What can Latin American governments do to help their most fragile populations during public health emergencies? Many Latin American governments expanded cash transfers to the poor and informal workers to sustain incomes and facilitate stay-at-home orders. These are commendable and necessary measures. However, precisely because these groups are often outside of the formal economy, not all the government help reached those most in need in a timely manner (Busso et al., 2020; Rauls, 2020). Moreover, many people who lack access to online banking had to wait in line for long hours, which increased their risk of exposure to COVID-19. In Peru, the economic support measures included four cash transfer programmes: Bono Yo Me Quedo en Casa, Bono Rural, Bono Independiente, and Bono Universal Familiar. While some of these transfers targeted self-employed and informal workers, the databases used were incomplete and outdated (Blofield et al., 2020; Jaramillo and López, 2021; OECD, 2020). In addition to the deficiencies in targeting and registering beneficiaries, the difficulties for an orderly and timely distribution of cash transfers meant that some households most in need did not receive help or did so only several months into the pandemic. Blofield et al. (2020: 51) show that only about 60 per cent of the informal population in Peru was reached by the emergency cash transfer programmes implemented during the pandemic, leaving an important coverage gap.

In addition to cash transfers, Latin American governments need to make sure that economically vulnerable people have access to food and essential medication during this period. Since the presence of state institutions is weak in the low-income areas of many Latin American countries (O’Donnell, 1993), governments should collaborate with social movements and grassroots actors to make sure that the vital needs of the economically vulnerable sectors of the population are met (Hummel et al., 2021; Zarazaga, 2020). The governments should also make sure that the informal workers who simply cannot stop working (e.g. food vendors in public markets) do so safely. Good quality personal protective equipment should be distributed widely among frontline informal workers who are in frequent contact with other people to prevent large outbreaks, such as the one that took place in Latin America’s largest market, the Mercado Central de Abasto in Mexico City (Sheridan, 2020). More broadly, Latin American governments should learn from the COVID-19 pandemic and develop rapid response plans detailing how they will assist their most economically vulnerable populations in future public health emergencies. The significant database updates that accompanied the welfare policy expansion efforts during the COVID-19 crisis (Blofield et al., 2020) should allow governments to respond more swiftly and effectively to the next pandemic or crisis.

## Supplemental Material

Online supplementary file 1 - Supplemental material for A Tale of Two Pandemics: Economic Inequality and Support for Containment Measures in Peru Click here for additional data file. Supplemental material, Online supplementary file 1, for A Tale of Two Pandemics: Economic Inequality and Support for Containment Measures in Peru by Miguel Carreras, Sofia Vera and Giancarlo Visconti in Journal of Politics in Latin America

## Notes

1. For a review of the varied social protection measures adopted in Latin America during the COVID-19 crisis, see Blofield et al. (2020).

2. Hundreds of people violating the curfew were arrested and issued fines: https://elperuano.pe/noticia-presidente-anuncia-multa-para-quienes-incumplan-cuarentena-por-coronavirus-94363.aspx

3. See Appendix B.

4. See questions in Appendix C in supplemental material.

5. These answers use the following scale: 1. *Strongly disagree*, 2. *Disagree*, 3. *Neither agree nor disagree*, 4. *Agree*, and 5. *Strongly agree*.

6. Because the lockdown restrictions were still in place.

7. In the case of the preference questions that take a five-point scale, we generate a binary indicator of agree or strongly agree with the main statement.

8. This item is based on the following question: “We know that people with certain diseases or pre-existing conditions are the most vulnerable to COVID-19 (i.e. those older than 65 and those with chronic diseases such as diabetes, hypertension, cancer, and cardiovascular or pulmonary conditions). Do you or someone in your home belong to this group?”
