# Supplementary material for: A Tale of Two Pandemics: Economic Inequality and Support for Containment Measures in Peru
Source: Journal of Politics in Latin America. 2021 Dec;13(3):358–75. doi: 10.1177/1866802X211035393 (PMC8361233; doi:10.1177/1866802X211035393)
Supplement: Online supplementary file 1 - Supplemental material for A Tale of Two Pandemics: Economic Inequality and Support for Containment Measures in Peru [file sj-pdf-1-pla-10.1177_1866802X211035393.pdf]

Supplementary Appendix for:

A Tale of Two Pandemics: Economic Inequality and Support for Containment Measures in Peru

Appendix A. Timeline of quarantine mandate in Peru

Appendix B. Inequalities in Peru

Appendix C. 2018/19 LAPOP Data

Appendix D: Questions used to construct the socioeconomic vulnerabilities indicator

Appendix A. Timeline

March 9: First positive case detected.

Start of Quarantine: The First "Hammer"

March 15: President announces nationwide state of emergency for 15 days.
March 16: President announces new measures.
March 17: Only holders of a "special pass" allowed to circulate in public.
March 18: President announces national curfew from 8 pm to 5 am.
March 19: First death confirmed.
March 26: Extension of national quarantine until April 12.
March 27: Creation of a national registry of curfew violators.
March 30: President announces that curfew now goes from 6 pm to 5 am in Lima; curfew in hardest hit regions to start at 4 pm (Loreto, Tumbes, Piura, La Libertad, Lambayeque).
March 30: Creation of a "working pass" to circulate in public, enforced by police.

New Measures: The Second "Hammer"

April 2: President announces gender-based quarantine.
April 3: Decree mandates the use of a facial mask.
April 8: Extension of national quarantine until April 26.
April 10: New curfew hours –from 6 pm to 4 am nationwide; but curfew in hardest hit regions to go from 4 pm to 4 am (Tumbes, Loreto, La Libertad, Piura, Lambayeque).
End of Second "Hammer"
April 11: Gender-based quarantine lifted. One person to do grocery shopping. Sundays everyone stays home.
April 13: Selective curfew hours –from 6pm nationwide but from 4 pm in La Libertad, Lambayeque, and Loreto.
April 14: The value of fines for breach of quarantine are announced (legislative decree).
April 15: Peru is the second LA country with more cases after Brazil.
April 19: Supermarkets and pharmacies to reopen.
April 23: Extension of national quarantine until May 10.
April 24: Day 40 of quarantine. Only essential businesses are allowed to operate.
April 29: Judiciary approves a protocol for resuming work in May 10.
April 30: Creation of a "vehicle pass" to circulate in public.

Reopening 1st Phase

May 2: Government announces reopening of economic activities in 4 phases. 22 activities will start operating again.
May 4: Experts question nationwide quarantine. Need intelligent or selective quarantine.
Mat 6: Labor minister announces sanitary protocols for firms resuming operations.
May 8: Extension of quarantine until May 24.
May 8: Production minister announces sanitary protocols for restaurant industry.
May 8: Housing minister announces sanitary protocols for construction industry.
May 12: Transport minister announces measures for public transportation users and drivers.

May 21-28: IEP survey.
May 22: Interior minister shows agreement with a focalized quarantine.
May 22: Extension of national quarantine until June 30; new curfew hours –starting now at 9 pm (until 4 am) with the exception of 8 regions.
May 22: Government announces the economic reopening will expand to new activities in the services and the commerce sectors.
May 23: Production minister announces sanitary protocol for delivery services.
May 23: Public sector to resume activities in May 25.
May 23: People in health risk group will not leave home (decree).
May 23: Particular vehicles to be allowed for circulation.

Appendix B. Inequality in Peru

Table A1 shows some simple descriptive statistics for services and amenities at home: refrigerator, bathroom, drinking water, computer, and internet service. The table also shows the proportion of informal workers as a share of the economically active population. All proportions by income groups are estimated with survey weights.

Table A1. Inequality in living and working conditions by income groups (LAPOP 2018)

Household Income	Refrigerator	Bathroom	Water	Computer	Internet	Informal
$0-$480 (N=359)	0.443	0.684	0.869	0.134	0.115	0.899
$481-$1000 (N=421)	0.630	0.798	0.869	0.310	0.279	0.777
$1000-$1800 (N=336)	0.764	0.890	0.938	0.563	0.527	0.646
More than $1800 (N=276)	0.906	0.949	0.967	0.772	0.724	0.459
All (N=1,392)	0.664	0.822	0.903	0.408	0.375	0.685

Table A2 shows some simple proportion of population living under overcrowded housing by levels of poverty. Data comes from the 2019 Peruvian Household Survey (ENAHO). According to ENAHO, an overcrowded housing is defined as one which has more than three persons per room. All proportions are estimated with survey weights.

Table A2. Population living in overcrowded households by poverty (ENAHO 2019)

	Proportion Overcrowding	Stand. Err. Overcrowding
National	0.041	0.002
Not Poor	0.026	0.001
Poor	0.113	0.006
Extreme poor	0.151	0.014

Appendix C. 2018/19 LAPOP Data

Below, we list the question wording of the variables used to construct table 1B.

Could you tell me if you have ... drinking water inside the house?
(1) Yes
(2) No

Could you tell me if you have ... a bathroom inside the house?
(1) Yes
(2) No

Could you tell me if you have ... a computer?
(1) Yes
(2) No

Could you tell me if you have ... a refrigerator inside the house?
(1) Yes
(2) No

Could you tell me if you have ... internet service inside the house?
(1) Yes
(2) No

In your primary occupation, are you…?
(1) Employed by the state or a state-owned firm
(2) Employed in the private sector
(3) Business owner or partner
(4) Own-account (self-employed) worker
(5) Worker without pay
(6) No response/Don't know

For this job, do you or your employer make contributions to your retirement?
(1) Si
(2) No

In which of the following ranges is the monthly family income of this household, including remittances from abroad and the income of all adults and children who they work?
(00) No income
(01) Less than 210 soles
(02) From 210 to 340 soles
(03) From 341 to 480 soles
(04) From 481 to 620 soles
(05) From 621 to 770 soles
(06) From 771 to 930 soles
(07) From 931 to 1000 soles
(08) From 1001 to 1100 soles

(09) From 1101 to 1200 soles
(10) From 1201 to 1400 soles
(11) From 1401 to 1600 soles
(12) From 1601 to 1800 soles
(13) From 1801 to 2100 soles
(14) From 2101 to 2700 soles
(15) From 2701 to 3000 soles
(16) More than 3000 soles
(888888) Doesn’t know
(988888) No response

Appendix D: Questions used to construct the socioeconomic vulnerabilities indicator

The *Instituto de Estudios Peruanos* uses the following eight questions to construct an eight-point scale of economic vulnerability.

Question 1: In order to group your answers with those of other people with similar characteristics to you, we would like you to answer the following questions regarding the head of the household. Head of household is the person, man or woman, aged 15 or over, who contributes the most economically at home or makes financial decisions for the family, and lives at this home. Home is the group of people who, living in the same house, prepare and consume their food together. What is the last year or degree of studies and level that the head of household passed?

1. No education / Early childhood education
2. Incomplete or complete Primary / Incomplete Secondary
3. Complete secondary / incomplete technical superior
4. Complete technical superior
5. Incomplete university superior
6. Complete university superior
7. Postgraduate university

Question 2: Is the head of the household affiliated with a private health insurance or EPS, other than ESSALUD or SIS?

1. No
2. Yes

Question 3: Which of these assets (in case they are working) do you have in your home?

Rows

1. Computer, laptop, tablet running
2. Washing machine in operation
3. Microwave oven in operation
4. Refrigerator / Freezer Running

Columns

1. No
2. Yes

Question 4: Which of these services do you have in your home?

Rows

1. Landline phone
2. Cable TV
3. Internet (without sharing by cell phone)
4. Cellular with Internet (cellular data) this is not used in the calculation

Columns

1. No
2. Yes

Question 5: Which of the following assets (in case they are working) do you have in your home?

Rows

1. Car and / or van only for private use (NOT TAXI OR COMPANY CAR)

2. Paid domestic service in the home (MINIMUM THAT YOU GO HOME ONCE A WEEK). Columns

1. No
2. Yes

Question 6: What is the predominant material in the floors of your home?

1. Dirt / other material (sand and raw planks)
2. Wood (decking) / tapestry
3. Unpolished or polished cement
4. Asphalt sheets or similar / vinyl, mosaic or similar / wood-type laminate
5. Tiles / terraces, majolica, ceramic
6. Parquet or polished wood and the like; porcelain tile, carpet, marble

Question 7: Is the predominant material of the walls brick or concrete block?

1. No
2. Yes

Question 8: The bathroom or toilet that you have in your home is CONNECTED to:

1. You do not have a bathroom / you are not connected to a public network.
2. Shared bathroom outside the house. (Ahem: fifths, corralones, rooms with shared bathroom, etc.)
3. Bathroom inside the house
